# 1-Cyclo­hexyl-6,7-dimeth­oxy-1,4-di­hydro­naphthalene

**DOI:** 10.1107/S1600536814010204

**Published:** 2014-05-17

**Authors:** Shao-Yuan Chen, Ya-Ping Zhang, Bing Huang, Jia-Jun Yu, Wei-Yong Shi

**Affiliations:** a Institute of Biotechnology, Zhejiang University, People’s Republic of China; b College of Pharmaceutical Science, Zhejiang University, People’s Republic of China; c College of Agriculture and Biotechnology, Zhejiang University, People’s Republic of China; d College of Environmental & Resource Sciences, Zhejiang University, People’s Republic of China

## Abstract

The title compound, C_18_H_24_O_2_, was isolated from the leaves extract of *Ficus carica* L. The cyclo­hexane ring displays a chair conformation whereas the cyclo­hexa-1,4-diene ring adopts a flattened boat conformation with methyl C atoms at the prow and stern. In the crystal, mol­ecules are linked by weak C—H⋯O hydrogen bonds into supra­molecular chains propagated along the *b*-axis direction.

## Related literature   

For the bioactivity of the title compound, see: Fang *et al.* (2008 ▶); Xie & Zhuang (2010 ▶). For biological activity of compounds isolated from *Ficus carica* L, see: Joseph & Raj (2011 ▶).
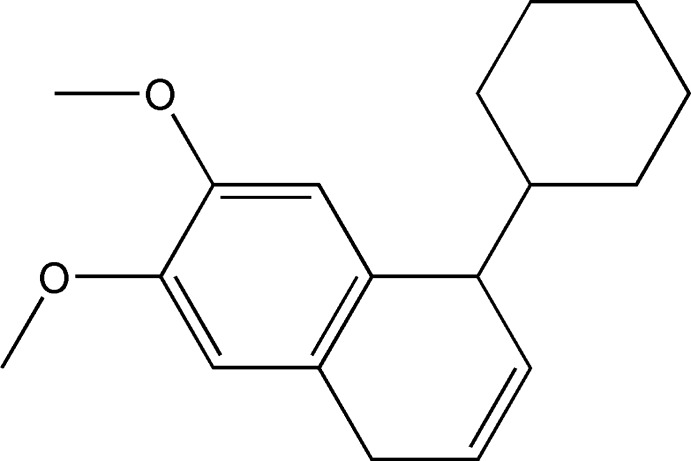


## Experimental   

### 

#### Crystal data  

C_18_H_24_O_2_
*M* *_r_* = 272.37
Monoclinic, 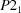

*a* = 9.0635 (4) Å
*b* = 6.3973 (3) Å
*c* = 13.9872 (7) Å
β = 104.241 (5)°
*V* = 786.08 (6) Å^3^
*Z* = 2
Cu *K*α radiation
μ = 0.57 mm^−1^
*T* = 294 K
0.28 × 0.15 × 0.12 mm

#### Data collection  

Agilent Xcalibur (Atlas, Gemini ultra) diffractometer
Absorption correction: multi-scan (*CrysAlis PRO*; Agilent, 2012 ▶) *T*_min_ = 0.76, *T*_max_ = 0.85
5062 measured reflections
2737 independent reflections
2318 reflections with *I* > 2σ(*I*)
*R*_int_ = 0.027

#### Refinement  

*R*[*F* ^2^ > 2σ(*F* ^2^)] = 0.039
*wR*(*F* ^2^) = 0.114
*S* = 1.04
2737 reflections
184 parameters
1 restraint
H-atom parameters constrained
Δρ_max_ = 0.10 e Å^−3^
Δρ_min_ = −0.10 e Å^−3^

### 

Data collection: *CrysAlis PRO* (Agilent, 2012 ▶); cell refinement: *CrysAlis PRO*; data reduction: *CrysAlis PRO*; program(s) used to solve structure: *SHELXTL* (Sheldrick, 2008 ▶); program(s) used to refine structure: *SHELXTL*; molecular graphics: *SHELXTL*; software used to prepare material for publication: *SHELXTL*.

## Supplementary Material

Crystal structure: contains datablock(s) I, global. DOI: 10.1107/S1600536814010204/xu5788sup1.cif

Structure factors: contains datablock(s) I. DOI: 10.1107/S1600536814010204/xu5788Isup2.hkl

Supporting information file. DOI: 10.1107/S1600536814010204/xu5788Isup3.cml

CCDC reference: 990756

Additional supporting information:  crystallographic information; 3D view; checkCIF report

## Figures and Tables

**Table 1:** Hydrogen-bond geometry (Å, °)

*D*—H⋯*A*	*D*—H	H⋯*A*	*D*⋯*A*	*D*—H⋯*A*
C18—H18*A*⋯O2^i^	0.96	2.59	3.272 (3)	129

Symmetry code: (i) 
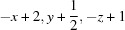
.

**Fig. 1.**
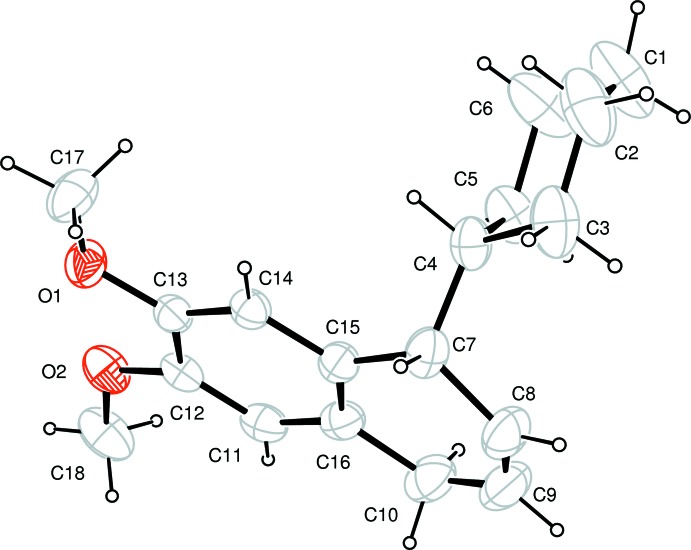
The molecular structure of (I) with 30% probability displacement ellipsoids (arbitrary spheres for H atoms).

## supplementary crystallographic information

### 1. Comment

Ficus carica *L*. is a deciduous tree belonging to the Moraceae family. Different biologically activity compounds have been isolated from this plant (Joseph & Raj, 2011). The leaf extracts of Ficus carica *L*. show the potential activity of inhibit the growth of the cancer cell, antioxidative and antibiosis (Xie & Zhuang, 2010; Fang *et al.*, 2008). The title compound is one of leaves extracts from Ficus carica *L*.

In the title compound, the cyclohexane ring displays the chair conformation whereas the cyclohexadiene ring adopts the flattened boat conformation with the methyl-C atoms (C7 and C10) on the prow and stern, respectively. In the crystal, the molecules are linked by weak C—H···O hydrogen bonds into the supramolecular chains running along the *b*-axis direction.

### 2. Experimental

The leaves of Ficus carica was be extracted by petroleum ether. The upper phase was filtered and evaporated *in vacuo* to obtain the crystals. The single crystals were recrystallized from a hexane solution.

### 3. Refinement

H atoms were placed in calculated positions with C—H = 0.93–0.98 Å, and refined in riding mode with *U*_iso_(H) = 1.5*U*_eq_(C) for methyl H atoms and 1.2*U*_eq_(C) for the others.

### Crystal data

**Table:**

C_18_H_24_O_2_	*F*(000) = 296
*M**_r_* = 272.37	*D*_x_ = 1.151 Mg m^−^^3^
Monoclinic, *P*2_1_	Melting point: 423 K
Hall symbol: P 2yb	Cu *K*α radiation, λ = 1.54180 Å
*a* = 9.0635 (4) Å	Cell parameters from 5062 reflections
*b* = 6.3973 (3) Å	θ = 3.2–67.6°
*c* = 13.9872 (7) Å	µ = 0.57 mm^−^^1^
β = 104.241 (5)°	*T* = 294 K
*V* = 786.08 (6) Å^3^	Needle, colourless
*Z* = 2	0.28 × 0.15 × 0.12 mm

### Data collection

**Table:**

Agilent Xcalibur (Atlas, Gemini ultra) diffractometer	2737 independent reflections
Radiation source: fine-focus sealed tube	2318 reflections with *I* > 2σ(*I*)
Graphite monochromator	*R*_int_ = 0.027
ω scans	θ_max_ = 67.6°, θ_min_ = 3.3°
Absorption correction: multi-scan (*CrysAlis PRO*; Agilent, 2012)	*h* = −10→10
*T*_min_ = 0.76, *T*_max_ = 0.85	*k* = −7→7
5062 measured reflections	*l* = −16→16

### Refinement

**Table:**

Refinement on *F*^2^	Secondary atom site location: difference Fourier map
Least-squares matrix: full	Hydrogen site location: inferred from neighbouring sites
*R*[*F*^2^ > 2σ(*F*^2^)] = 0.039	H-atom parameters constrained
*wR*(*F*^2^) = 0.114	*w* = 1/[σ^2^(*F*_o_^2^) + (0.0621*P*)^2^ + 0.0278*P*] where *P* = (*F*_o_^2^ + 2*F*_c_^2^)/3
*S* = 1.04	(Δ/σ)_max_ < 0.001
2737 reflections	Δρ_max_ = 0.10 e Å^−^^3^
184 parameters	Δρ_min_ = −0.10 e Å^−^^3^
1 restraint	Extinction correction: *SHELXTL* (Sheldrick, 2008), Fc^*^=kFc[1+0.001xFc^2^λ^3^/sin(2θ)]^-1/4^
Primary atom site location: structure-invariant direct methods	Extinction coefficient: 0.0191 (17)

### Special details

**Table:**

Geometry. All e.s.d.'s (except the e.s.d. in the dihedral angle between two l.s. planes) are estimated using the full covariance matrix. The cell e.s.d.'s are taken into account individually in the estimation of e.s.d.'s in distances, angles and torsion angles; correlations between e.s.d.'s in cell parameters are only used when they are defined by crystal symmetry. An approximate (isotropic) treatment of cell e.s.d.'s is used for estimating e.s.d.'s involving l.s. planes.
Refinement. Refinement of *F*^2^ against ALL reflections. The weighted *R*-factor *wR* and goodness of fit *S* are based on *F*^2^, conventional *R*-factors *R* are based on *F*, with *F* set to zero for negative *F*^2^. The threshold expression of *F*^2^ > σ(*F*^2^) is used only for calculating *R*-factors(gt) *etc*. and is not relevant to the choice of reflections for refinement. *R*-factors based on *F*^2^ are statistically about twice as large as those based on *F*, and *R*- factors based on ALL data will be even larger.

### Fractional atomic coordinates and isotropic or equivalent isotropic displacement parameters (Å^2^)

**Table:**

	*x*	*y*	*z*	*U*_iso_*/*U*_eq_	
O1	0.67571 (16)	0.5524 (2)	0.46174 (9)	0.0672 (4)	
O2	0.83494 (17)	0.8807 (2)	0.52180 (12)	0.0718 (4)	
C1	0.2221 (5)	0.3458 (6)	0.8467 (4)	0.1353 (15)	
H1A	0.2527	0.3728	0.9170	0.162*	
H1B	0.1118	0.3393	0.8273	0.162*	
C2	0.2869 (5)	0.1400 (6)	0.8249 (3)	0.1189 (12)	
H2A	0.2463	0.1053	0.7559	0.143*	
H2B	0.2559	0.0314	0.8642	0.143*	
C3	0.4597 (4)	0.1463 (4)	0.8472 (2)	0.0911 (8)	
H3A	0.4972	0.0133	0.8296	0.109*	
H3B	0.5010	0.1678	0.9173	0.109*	
C4	0.5140 (3)	0.3217 (3)	0.78985 (15)	0.0649 (5)	
H4	0.4671	0.2958	0.7199	0.078*	
C5	0.4514 (3)	0.5281 (4)	0.8149 (2)	0.0806 (6)	
H5A	0.4832	0.6389	0.7771	0.097*	
H5B	0.4921	0.5582	0.8843	0.097*	
C6	0.2766 (4)	0.5223 (6)	0.7919 (3)	0.1258 (13)	
H6A	0.2393	0.6543	0.8107	0.151*	
H6B	0.2357	0.5039	0.7215	0.151*	
C7	0.6876 (3)	0.3242 (3)	0.80090 (15)	0.0650 (5)	
H7	0.7156	0.1848	0.7823	0.078*	
C8	0.7775 (3)	0.3614 (5)	0.90618 (18)	0.0876 (8)	
H8	0.7745	0.2588	0.9528	0.105*	
C9	0.8597 (3)	0.5300 (6)	0.93588 (19)	0.0947 (9)	
H9	0.9077	0.5409	1.0026	0.114*	
C10	0.8806 (3)	0.7015 (5)	0.87079 (18)	0.0859 (8)	
H10A	0.9882	0.7343	0.8837	0.103*	
H10B	0.8287	0.8247	0.8865	0.103*	
C11	0.8579 (2)	0.7884 (3)	0.69386 (17)	0.0616 (5)	
H11	0.9169	0.9059	0.7159	0.074*	
C12	0.8086 (2)	0.7530 (3)	0.59475 (15)	0.0552 (5)	
C13	0.72044 (19)	0.5754 (3)	0.56185 (13)	0.0501 (4)	
C14	0.6851 (2)	0.4408 (3)	0.62918 (14)	0.0514 (4)	
H14	0.6274	0.3224	0.6068	0.062*	
C15	0.7338 (2)	0.4775 (3)	0.73125 (14)	0.0539 (5)	
C16	0.8218 (2)	0.6528 (3)	0.76285 (14)	0.0610 (5)	
C17	0.5812 (3)	0.3808 (4)	0.42523 (17)	0.0823 (7)	
H17A	0.5560	0.3826	0.3544	0.123*	
H17B	0.4896	0.3895	0.4478	0.123*	
H17C	0.6336	0.2532	0.4485	0.123*	
C18	0.9424 (3)	1.0441 (4)	0.5498 (2)	0.0919 (8)	
H18A	0.9578	1.1121	0.4919	0.138*	
H18B	1.0373	0.9872	0.5870	0.138*	
H18C	0.9051	1.1436	0.5896	0.138*	

### Atomic displacement parameters (Å^2^)

**Table:**

	*U*^11^	*U*^22^	*U*^33^	*U*^12^	*U*^13^	*U*^23^
O1	0.0746 (9)	0.0747 (10)	0.0547 (7)	−0.0196 (8)	0.0205 (6)	−0.0016 (7)
O2	0.0705 (8)	0.0596 (9)	0.0941 (11)	−0.0131 (7)	0.0367 (7)	0.0036 (8)
C1	0.136 (3)	0.106 (3)	0.203 (4)	−0.003 (2)	0.117 (3)	0.007 (3)
C2	0.154 (3)	0.091 (2)	0.141 (3)	−0.035 (2)	0.092 (2)	−0.010 (2)
C3	0.140 (2)	0.0570 (13)	0.0913 (17)	−0.0017 (15)	0.0577 (16)	0.0064 (12)
C4	0.0840 (13)	0.0572 (11)	0.0592 (11)	0.0032 (10)	0.0287 (10)	0.0082 (9)
C5	0.0939 (16)	0.0562 (12)	0.1033 (17)	0.0071 (12)	0.0465 (13)	0.0113 (12)
C6	0.097 (2)	0.102 (2)	0.202 (4)	0.015 (2)	0.081 (2)	0.028 (3)
C7	0.0811 (13)	0.0583 (11)	0.0562 (11)	0.0179 (11)	0.0183 (9)	0.0064 (9)
C8	0.0953 (17)	0.103 (2)	0.0590 (13)	0.0274 (17)	0.0093 (12)	0.0216 (14)
C9	0.0919 (18)	0.123 (3)	0.0570 (12)	0.0246 (19)	−0.0047 (12)	−0.0068 (17)
C10	0.0874 (16)	0.094 (2)	0.0661 (14)	0.0091 (15)	0.0004 (11)	−0.0205 (14)
C11	0.0474 (9)	0.0562 (12)	0.0812 (13)	−0.0035 (8)	0.0157 (9)	−0.0141 (10)
C12	0.0431 (8)	0.0527 (10)	0.0743 (12)	0.0006 (8)	0.0229 (8)	−0.0012 (9)
C13	0.0449 (9)	0.0532 (10)	0.0545 (10)	−0.0011 (7)	0.0169 (7)	−0.0041 (8)
C14	0.0488 (9)	0.0498 (10)	0.0570 (10)	−0.0028 (8)	0.0157 (8)	−0.0043 (8)
C15	0.0546 (10)	0.0544 (11)	0.0532 (10)	0.0115 (8)	0.0140 (8)	−0.0018 (8)
C16	0.0533 (10)	0.0637 (12)	0.0623 (11)	0.0095 (9)	0.0070 (9)	−0.0139 (10)
C17	0.0982 (16)	0.0818 (16)	0.0584 (12)	−0.0295 (14)	0.0032 (11)	−0.0050 (11)
C18	0.0838 (16)	0.0603 (14)	0.146 (2)	−0.0186 (13)	0.0553 (16)	−0.0067 (15)

### Geometric parameters (Å, º)

**Table:**

O1—C13	1.367 (2)	C7—C8	1.516 (3)
O1—C17	1.409 (3)	C7—H7	0.9800
O2—C12	1.373 (2)	C8—C9	1.318 (4)
O2—C18	1.417 (3)	C8—H8	0.9300
C1—C2	1.503 (5)	C9—C10	1.468 (5)
C1—C6	1.514 (5)	C9—H9	0.9300
C1—H1A	0.9700	C10—C16	1.505 (3)
C1—H1B	0.9700	C10—H10A	0.9700
C2—C3	1.520 (5)	C10—H10B	0.9700
C2—H2A	0.9700	C11—C12	1.367 (3)
C2—H2B	0.9700	C11—C16	1.395 (3)
C3—C4	1.529 (3)	C11—H11	0.9300
C3—H3A	0.9700	C12—C13	1.400 (3)
C3—H3B	0.9700	C13—C14	1.371 (3)
C4—C5	1.512 (3)	C14—C15	1.406 (3)
C4—C7	1.543 (3)	C14—H14	0.9300
C4—H4	0.9800	C15—C16	1.384 (3)
C5—C6	1.537 (4)	C17—H17A	0.9600
C5—H5A	0.9700	C17—H17B	0.9600
C5—H5B	0.9700	C17—H17C	0.9600
C6—H6A	0.9700	C18—H18A	0.9600
C6—H6B	0.9700	C18—H18B	0.9600
C7—C15	1.512 (3)	C18—H18C	0.9600
			
C13—O1—C17	117.13 (16)	C4—C7—H7	106.8
C12—O2—C18	117.82 (19)	C9—C8—C7	124.2 (2)
C2—C1—C6	111.0 (3)	C9—C8—H8	117.9
C2—C1—H1A	109.4	C7—C8—H8	117.9
C6—C1—H1A	109.4	C8—C9—C10	124.4 (2)
C2—C1—H1B	109.4	C8—C9—H9	117.8
C6—C1—H1B	109.4	C10—C9—H9	117.8
H1A—C1—H1B	108.0	C9—C10—C16	113.5 (2)
C1—C2—C3	111.7 (3)	C9—C10—H10A	108.9
C1—C2—H2A	109.3	C16—C10—H10A	108.9
C3—C2—H2A	109.3	C9—C10—H10B	108.9
C1—C2—H2B	109.3	C16—C10—H10B	108.9
C3—C2—H2B	109.3	H10A—C10—H10B	107.7
H2A—C2—H2B	107.9	C12—C11—C16	121.65 (18)
C2—C3—C4	111.1 (2)	C12—C11—H11	119.2
C2—C3—H3A	109.4	C16—C11—H11	119.2
C4—C3—H3A	109.4	C11—C12—O2	125.58 (18)
C2—C3—H3B	109.4	C11—C12—C13	119.05 (17)
C4—C3—H3B	109.4	O2—C12—C13	115.35 (16)
H3A—C3—H3B	108.0	O1—C13—C14	125.16 (16)
C5—C4—C3	109.39 (18)	O1—C13—C12	115.19 (15)
C5—C4—C7	113.54 (19)	C14—C13—C12	119.65 (16)
C3—C4—C7	114.01 (19)	C13—C14—C15	121.65 (17)
C5—C4—H4	106.4	C13—C14—H14	119.2
C3—C4—H4	106.4	C15—C14—H14	119.2
C7—C4—H4	106.4	C16—C15—C14	118.14 (17)
C4—C5—C6	110.9 (3)	C16—C15—C7	123.28 (18)
C4—C5—H5A	109.5	C14—C15—C7	118.58 (17)
C6—C5—H5A	109.5	C15—C16—C11	119.85 (17)
C4—C5—H5B	109.5	C15—C16—C10	121.5 (2)
C6—C5—H5B	109.5	C11—C16—C10	118.7 (2)
H5A—C5—H5B	108.0	O1—C17—H17A	109.5
C1—C6—C5	111.1 (3)	O1—C17—H17B	109.5
C1—C6—H6A	109.4	H17A—C17—H17B	109.5
C5—C6—H6A	109.4	O1—C17—H17C	109.5
C1—C6—H6B	109.4	H17A—C17—H17C	109.5
C5—C6—H6B	109.4	H17B—C17—H17C	109.5
H6A—C6—H6B	108.0	O2—C18—H18A	109.5
C15—C7—C8	110.8 (2)	O2—C18—H18B	109.5
C15—C7—C4	112.31 (16)	H18A—C18—H18B	109.5
C8—C7—C4	112.98 (18)	O2—C18—H18C	109.5
C15—C7—H7	106.8	H18A—C18—H18C	109.5
C8—C7—H7	106.8	H18B—C18—H18C	109.5

### Hydrogen-bond geometry (Å, º)

**Table:**

*D*—H···*A*	*D*—H	H···*A*	*D*···*A*	*D*—H···*A*
C18—H18*A*···O2^i^	0.96	2.59	3.272 (3)	129

Symmetry code: (i) −*x*+2, *y*+1/2, −*z*+1.
